# Stable patterns, shifting risks: the impact of British Columbia’s decriminalization and recriminalization policies on drug use behaviours

**DOI:** 10.1186/s12954-025-01322-9

**Published:** 2025-10-14

**Authors:** Farihah Ali, Jordan Mende-Gibson, Cayley Russell, Savannah Torres-Salbach, Geoff Bardwell, Matthew Bonn, Juls Budau, Andrew Ivsins, Jürgen Rehm

**Affiliations:** 1 Institute for Mental Health Policy Research, Centre for Addiction and Mental Health (CAMH), 33 Ursula Franklin St., Toronto, ON M5S 2S1 Canada; 2 Ontario Node, Canadian Research Initiative in Substance Matters (CRISM), 33 Ursula Franklin St., Toronto, ON M5S 2S1 Canada; 3 Division of Social Behavioural Health Sciences, Dalla Lana School of Public Health, University of Toronto, 155 College Street, Toronto, ON M5T 3M6 Canada; 4 Faculty of Applied Health and Community Studies, School of Applied Health, Clinical Research, Sheridan College, 7899 McLaughlin Rd, Brampton, ON L6Y 0P8 Canada; 5 School of Public Health Sciences, University of Waterloo, Waterloo, ON Canada; 6 ChangeMark Research and Evaluation, Vancouver, BC Canada; 7 Canadian Collaboration for Prison Health and Education, School of Population and Public Health, University of British Columbia, 2206 East Mall, Vancouver, BC V6T1Z3 Canada; 8 British Columbia Centre On Substance Use, 400-1045 Howe Street, Vancouver, BC V6Z 2A9 Canada; 9 Department of Medicine, University of British Columbia, 317 - 2194 Health Sciences Mall, Vancouver, BC V6T 1Z3 Canada; 10 Campbell Family Mental Health Research Institute, Centre for Addiction and Mental Health (CAMH), 1001 Queen St. West, Toronto, ON M6J 1H4 Canada; 11 Department of Psychiatry Dalla Lana School of Public Health & Institute of Medical Science (IMS), University of Toronto, 1 King’s College Circle, Toronto, ON M5S 1A8 Canada; 12 Department of Psychiatry and Psychotherapy, Center for Interdisciplinary Addiction Research (ZIS), University Medical Center Hamburg-Eppendorf (UKE), Martinistr. 52, 20246 Hamburg, Germany; 13 Institute of Health Policy, Management and Evaluation, University of Toronto, 155 College St. 4th Floor, Toronto, ON M5T 3M6 Canada; 14 Institute of Medical Science, University of Toronto, 2374, 1 King’s College Circle, Toronto, Ontario M5S 1A8 Canada

**Keywords:** Canada, Drug policy, Decriminalization, People who use drugs, Recriminalization, Opioid use

## Abstract

**Background:**

Canada’s historical reliance on criminal justice approaches to drug policy has intensified structural and social stigma, and high-risk behaviours among people who use drugs. In response to pressure from local advocates, British Columbia implemented a pilot decriminalization policy in January 2023, permitting adults to possess up to 2.5 g of specified unregulated substances, cumulatively. While not designed to address the toxic drug supply directly, it aimed to reduce stigma and encourage engagement with health and harm reduction services. In May 2024, however, drug possession in public spaces was recriminalized, raising concerns about a return to punitive environments. To date, little is known about how these policy shifts have been experienced by people who use drugs themselves. We conducted a qualitative study exploring the impacts of British Columbia’s decriminalization policy and its subsequent recriminalization amendment on the drug use behaviours of people who use drugs across the province.

**Methods:**

A cross-sectional qualitative study with 75 people who use drugs across British Columbia, including a socio-demographic survey, and semi-structured interviews. Interviews were transcribed verbatim and analyzed using thematic analysis. The codebook was applied across all transcripts using a comparative approach to identify recurring patterns, divergent experiences, and key themes related to drug use behaviours.

**Results:**

Participants reported little to no change in their drug use patterns following either decriminalization or recriminalization, as drug use was primarily driven by dependence, routine, and structural factors. Nonetheless, many described a psychological benefit under decriminalization, including reduced shame, internalized stigma, and fear of criminalization. These gains were largely reversed following the recriminalization amendment, which pushed drug use back into hidden, high-risk environments. Participants also noted destabilizing shifts in the drug supply, including increased potency and a rise in less experienced dealers, linked to the 2.5 g threshold.

**Conclusion:**

Decriminalization did not significantly alter drug use behaviours but offered notable psychological relief for participants. The subsequent recriminalization amendment then reversed these perceived gains, illustrating how this abrupt policy change led to unintended consequences, undermining the original goals of the decriminalization policy. These findings highlight the need for sustained and structurally supported effective policy approaches that center the lived realities of people who use drugs.

## Background

British Columbia (BC) has long been at the forefront of Canada’s response to the overdose crisis, which has resulted in a staggering and sustained loss of life over the past decade [1]. Since a public health emergency was declared in 2016, the overdose crisis has claimed the lives of approximately 16,000 BC residents [1]. Like many jurisdictions, Canada’s historical approach to drug policy has been rooted in a punitive, enforcement-based model that treats substance use primarily as a criminal justice issue rather than a public health matter [2]. This framework has produced significant harms for people who use drugs, who consistently face disproportionate surveillance, criminalization, and structural discrimination [3]. Experiences of criminalization vary across contexts, as individuals may also be targeted based on their socio-demographic characteristics, such as their gender, race, or housing status [4, 5]. Marginalized groups, such as individuals experiencing homelessness, are especially vulnerable to police targeting and drug-related harms, the legal system broadly affects all people who use drugs by reinforcing stigma and exacerbating criminalization, which deters people who use drugs from accessing essential health and social services and drives drug use into hidden and isolated settings to avoid police detection and stigma [4, 5]. However, enforcement efforts are not uniformly experienced across the province and may differ depending on individuals location of residence [4]. For many, criminalization has also contributed to high-risk behaviours, such as needle reuse and rushing injections [5, 6], which can lead to injection infections [7]. Criminalization also reinforces the ideology that drug use is a morally wrong personal choice, further perpetrating the stigma that people who use drugs face [3].

To reduce these harms and address the overdose crisis, BC implemented a province-wide drug decriminalization pilot policy through a three-year exemption to the *Controlled Drugs and Substances Act.* This exemption came into effect on January 31, 2023 and is set to end on January 31, 2026 [8]. Under this policy, adults aged 18 and older are permitted to possess or carry up to a cumulative total of 2.5 g (g) of specified substances— opioids, cocaine, methamphetamine, and MDMA—for personal use without being arrested, charged, or have their drugs seized.

However, the decriminalization policy is limited in scope, and a number of activities remain criminalized. For instance, carrying more than 2.5 g of any combined substance remains criminalized. There are a number of substances not included under the policy as well which remain criminalized in any amount, such as benzodiazepines, a contaminant increasingly found in the province’s illicit opioid supply [8, 9]. The decriminalization policy only applies to adults, meaning that youth under 18 are subject to criminalization. In addition, possession for the purpose of selling, distribution, or trafficking, remains criminalized [8]. Finally, police are still able to enforce against drug use in public spaces, by seizing drugs and arresting people who use drugs when required, as the act of using drugs is not included under the scope of the policy [8].

The primary goals of the decriminalization policy are to reduce the stigma associated with drug use and encourage engagement with overdose prevention services, both of which can prevent people who use drugs from using alone, which significantly increases the risk of fatal overdoses [10]. Using drugs alone also puts people who use drugs at increased risk of physical and sexual violence [11]. Provincial organizations explicitly acknowledged the dangers of solitary drug use, where the absence of bystanders eliminates the possibility of naloxone administration or timely emergency response [10]. Although the policy does not directly address the toxic and the increasingly unpredictable drug supply, it is intended to create safer conditions for people who use drugs. By removing criminal penalties for possession under 2.5 g, the policy aims to reduce the fear of arrest, foster safer drug use practices, and mitigate the broader harms of criminalization for personal possession [10]. Despite these aims, questions quickly emerged regarding the practical impacts of the policy on the daily lives of people who use drugs. Previous research has suggested that most people who use drugs reported little to no change in their drug use patterns, drug carrying practices, or service engagement following decriminalization [12–15]. These findings prompted further questions about the degree to which decriminalization has translated into meaningful change in the lives of people who use drugs.

Further complicating the policy’s potential impact, in May 2024, BC amended the policy to prohibit drug possession for personal use in public spaces, effectively recriminalizing many of the locations where people who use drugs commonly carry and consume drugs, particularly among those experiencing homelessness [10, 16]. This amendment to the policy (referred to from herein as ‘recriminalization’) raises additional concerns about increased police surveillance, social displacement, and the potential return to unsafe environments and drug use practices, particularly for individuals experiencing homelessness who rely on public spaces for drug use. These concerns suggest that this policy amendment undermines the initial goals of decriminalization [17, 18].

As the policy landscape continues to shift, it is crucial to understand how both decriminalization and the subsequent recriminalization amendment are being experienced by people who use drugs, and how these policy shifts have impacted drug use behaviours among people who use drugs. Direct engagement with people most affected by these changes is essential to accurately evaluate their real-world outcomes, and the application of the policy. Our initial research presented findings from the first year of the decriminalization policy [12]. This study revealed that participants’ drug use patterns remained largely unchanged, with a smaller number of participants adjusting their carrying amount to less than 2.5 g to avoid criminalization. Building on our initial research [12], the present study specifically explores the lived experiences, perspectives, and behavioural impacts of both the decriminalization policy and the recriminalization amendment. This study offers important insights into how these evolving policies are shaping drug use practices, purchasing and carrying patterns, and personal safety for people who use drugs over time. Centering the voices of people who use drugs is essential to informing future drug policy development, enhancing harm reduction strategies, and supporting more effective responses to the ongoing overdose crisis in BC and beyond for future drug crises.

## Methods

### Study design and recruitment

We conducted a cross-sectional qualitative study, recruiting people who use drugs from across BC, including individuals from both rural and urban regions between February 20th, 2025 and April 8th, 2025. By including participants from across the province, we hoped to capture unique and diverse experiences from individuals residing in both rural/remote locations as well as urban centres. Participants were recruited via several avenues including both purposive and snowball sampling techniques [19, 20]. Purposive sampling utilized our established networks (consisting of harm reduction sites, drug user advocacy groups, academic researchers, and the project’s working group), who shared study information to individuals who met the eligibility criteria and circulated study flyers through their professional and social networks. Participants were also encouraged to share information about the study to potential participants through a snowball sampling approach. Through these sampling methods, we aimed to include a diverse range of participants who varied in age, gender, ethnicity, sexual orientation, and drug use patterns. Study recruitment continued until data saturation was achieved and no new themes emerged.

This study is part of a national evaluation of BC’s illicit drug decriminalization policy, funded by the Canadian Institutes for Health Research (CIHR; grant # EVD-184698), undertaken by the Ontario Node of the Canadian Research Initiative in Substance Matters (CRISM).

### Eligibility criteria

Prospective participants contacted the research team via a toll-free study line or email. Upon initial contact, a research team member screened participants for eligibility and scheduled an interview if eligible. Participant eligibility included: (1) resident of BC prior to January 31, 2023, (2) aged 18 years or older, (3) access to a telephone or the internet, (4) English-speaking, and (5) use of illegal drugs at least three times a week. Participants location of residence and use and frequency of illegal drugs was based on self-report. Proof of residency was not requested as participants may not have had valid identification or may not have wanted to disclose their address due to privacy concerns. Eligibility screening was conducted through an interviewer-administered process using Research Electronic Data Capture (REDCap), a secure web-based data collection software [21].

### Data collection

All participants provided informed consent prior to participating in the study, captured remotely via REDCap, and all interviews were conducted over the phone.

The interview process consisted of two parts: (1) a socio-demographic survey, and (2) a qualitative, semi-structured interview. The socio-demographic survey captured indicators such as location of residence, age, gender, housing status, drug use (e.g., types of drug(s) used, routes of administration, frequency of use), and overdose history. Survey responses were directly recorded in REDCap, by the interviewer.

The interview guide was developed collaboratively with the study’s working group, ensuring questions were relevant and would facilitate meaningful conversation. The guide included questions that explored patterns of drug use before and after decriminalization and recriminalization, including how much they used and carried, where and how often they used drugs, how they purchased drugs, and any changes in drug type or method of use. The interviews also probed the influence of policy changes on these behaviors, with specific questions about the impacts of the May 2023 amendment re-criminalizing drug use in public settings.

The interviews were conducted by a university-trained research team member (STS). Interviews were audio-recorded and were approximately 45 min in length. All participants received a $50.00 cash honorarium, distributed via online banking e-transfer or MoneyGram. Participants who did not have online banking options or valid ID to receive a MoneyGram were reimbursed through a harm reduction provider or peer who collected and distributed the honorarium on their behalf. Audio recordings were transcribed by an external transcription service.

### Data analysis and synthesis

#### Quantitative analysis

All socio-demographics and drug use survey data collected in REDCap were exported to a Microsoft Excel file. Basic frequency counts and percentages were calculated. Participant data were organized by age, gender, housing status and location. For housing status, those living in a private residence (alone or with others) were categorized as “Housed”, while participants without a regular place to stay were categorized as “Experiencing Homelessness”. Participants who noted living in temporary, transient housing, or other housing types (i.e. living in a harm reduction site, trailer) were categorized as “Experiencing Precarious Housing”. For location, participants were categorized as residing in either “Urban” or “Rural” locations based on BC’s Community Health Service Areas (CHSA) boundary configuration [22]. This classification system organizes BC’s land areas on a six-point scale based on level of urbanization (1-Metropolitan, 6-Remote). For reporting purposes, participants from areas classified as Metropolitan to Medium Urban were categorized as “Urban” and areas classified as Small Urban to Remote were categorized as “Rural”.

#### Qualitative analysis

During the data collection period, we debriefed as a team consistently to discuss the data, including assessments of data saturation. We paused recruitment in specific geographic regions once no new insights were emerging. We continued this process until we had reached overall data saturation for each region. Once collection was completed, all interview transcripts were reviewed by a research team member (STS) to ensure accuracy in the transcribing and were imported into NVivo (Version 15) for qualitative data analysis. An initial codebook was developed based on the research questions and preliminary insights gathered from the interviews. Reflexive notes were taken during and after interviews, including general reflections and takeaways of the interview interpreted by the researcher [23], followed by team debrief discussions which further guided codebook development. The codebook was created using a hierarchical structure to manage the complexity of data and allow for in-depth analysis. This included broad, overarching, parent codes (e.g., drug use behaviours and risks), which were then broken down into more specific child codes (e.g., changes in location of use, changes in frequency of use, changes in types of drugs used, etc.). Additionally, child codes were further refined into grandchild codes to capture detailed variations in behaviours (e.g., increases versus decreases in frequency of drug use, specific drugs used, etc.). This tiered coding framework facilitated both high-level thematic analysis and fine-grained examination of patterns across participants’ experiences. The research team then coded a subset of interviews during which the codebook was iteratively refined. During this process, new codes were created and existing codes were revised, ensuring clarity in the codes and that the codebook was able to generate meaningful analyses. Any discrepancies in the initial coding were resolved during team discussions. The final codebook was applied to all transcripts by one team member (STS) to ensure consistency in the coding process. An additional coder with lived experience independently coded a subset of transcripts to ensure coding was reflective of participants’ experiences. Any discrepancies were resolved during team discussions.

Once coding was complete, a consolidated “Master File” was developed in Word and circulated among the research team. The document provided a summary of all parent, child, and grandchild codes, noted the number of participants who discussed each code, and included illustrative quotes. To ensure comprehensive theme identification, the Master File was cross-referenced with reflexive notes taken during and after the interviews. Thematic analysis was conducted following Braun and Clarke’s approach [24, 25], with multiple rounds of coding using both inductive and deductive approaches to identify broader and smaller, more specific themes.

Specifically, we took a pragmatic epistemological stance to examine the real-world, lived experiences of people who use drugs amid the decriminalization and subsequent recriminalization policy changes [26]. In doing so, we hope these findings will inform future policy development that effectively address the health and social outcomes of people who use drugs.

### Participant autonomy and ethical considerations

No participants withdrew from the study due to ethical concerns; however, all were informed of their right to do so at any point. Given the sensitive nature of some interview questions, a number of participants chose to skip specific questions they were uncomfortable answering, in accordance with the voluntary and participant-centred approach of our study.

The study was approved by the CAMH Research Ethics Board (REB; #2023/088).

## Results

### Socio-demographic characteristics

A total of 126 participants contacted the toll-free study line or email, of whom 119 (94%) were eligible to participate. Among these, 18 (15%) were lost to follow-up, another 18 (15%) were excluded due to paused recruitment in specific geographic regions where data saturation had been reached, and 8 (7%) were pending interviews when overall data saturation was achieved.

A total of 75 people who use drugs participated in this study, located across the five Regional Health Authorities. Specifically, 19 (25%) participants resided in Vancouver Coastal Health Authority, 15 (20%) resided in Island Health Authority, 12 (16%) in Interior Health Authority, nine (12%) in Northern Health Authority, and seven (9%) in Fraser Health Authority. The majority (n = 45; 60%) of participants resided in urban regions and 30 (40%) resided in rural regions.

The average age of participants was 45 years (SD $\pm$ 10.35). The majority of participants were white (n = 52; 69%), cis men (n = 38; 51%). Approximately one third of participants (n = 24; 32%) had secondary or high school education, 22 (29%) participants had completed college/university, 12 (16%) had some college/university education, and three (4%) had vocational/trade school education. Refer to Table 1 for full details on participants socio-demographic characteristics.

**Table 1:** Participants’ socio-demographic characteristics (n = 75)

Socio-demographic characteristics	N	%
*Gender*		
Cis man	38	51
Cis woman	34	45
Non-binary	2	3
Transgender woman	1	1
*Age*		
20–29	6	8
30–39	16	21
40–49	27	36
50–59	17	23
60 +	6	8
Ethnicity*		
White (European descent)	52	69
Metis	11	15
First nations	11	13
Inuit	2	3
Black (African/Afro-Caribbean/African-Canadian descent)	2	3
South Asian (East Indian/Pakistani/Bangladeshi/Sri Lankan/Indo-Caribbean)	2	3
Latino (Latin American/Hispanic descent)	2	3
East/Southeast Asian (Chinese/Korean/Japanese/Filipino/Vietnamese/Thai)	2	3
Pacific islander	1	1
Status native	1	1
*Highest level of education*		
Secondary/high school	24	32
Some college/university	12	16
Vocational/trade school	3	4
College/university	22	29
*Housing status (last 30 days)*		
Housed (alone or with others)	38	51
Precarious housing (hotels, motels, single room occupancy [SRO], social/supportive/transitional housing, shelter)	21	28
Experiencing homelessness (houseless, couch surfing, no fixed address)	16	21
*Employment status*		
Employed (part-time or full-time)	25	33
On disability	21	28
Unemployed	18	24
Volunteer	9	12
*Regional health authority*		
Vancouver coastal	19	25
Island	15	20
Interior	12	16
Northern	9	12
Fraser	7	9
*Geographic location*		
Urban	45	60
Rural	30	40

*Participants were able to select more than one option

### Drug use profiles

The majority of participants (n = 62; 83%) reported daily drug use, with smaller proportions using every other day (n = 10; 13%) or a few times a week (n = 3; 4%). Methamphetamine was the most commonly used drug (n = 39; 52%), followed by crack-cocaine (n = 35; 47%) and illegal/street opioids (n = 33; 44%). The majority of participants were polysubstance users (n = 51; 68%). Inhalation was the predominant route of administration (n = 68; 91%), followed by injection (n = 17; 13%), and oral (n = 16; 12%). Refer to Table 2 for details on participants’ drug use profiles.

**Table 2:** Participant drug use profiles (n = 75)

Drug use patterns and characteristics	N	%
*Frequency of use*		
Daily	62	83
Every other day	10	13
A few (2–3) times a week	3	4
Drugs used*		
Methamphetamine	39	52
Crack-cocaine	35	47
Illegal/street opioids	33	44
Powder cocaine	14	19
Psychedelics/hallucinogens/dissociatives/sedatives/tranquilizers	10	13
Ecstasy/MDMA	5	7
Non-prescribed benzodiazepines	2	3
Non-prescribed opioids	1	1
Stimulant and opioid combinations	1	1
Other**	1	1
Polysubstance use	51	68
*Route of administration*		
Inhalation	68	91
Injection	17	13
Oral	16	12
Nasal	15	11

*Participants were able to select more than one option

**K-crack (a combination of ketamine and crack-cocaine

Within the six-month period prior to being surveyed, a total of 25 (33%) participants reported experiencing an overdose. Nearly a quarter (n = 17; 23%) experienced an overdose from consumption of “down,” which is the colloquial term for illicitly purchased fentanyl while seven (9%) experienced a benzodiazepine or tranquilizer-related overdose. A smaller number (n = 2; 3%) experienced a stimulant overdose.

### Qualitative findings

The interviews provided rich insights into how patterns of carrying, purchasing and using drugs were affected by BC’s decriminalization policy and its subsequent recriminalization amendment. These findings are organized into four thematic sub-sections: (a) Enduring Habits: Drivers of Drug Use Amid Policy Change, (b) Stable Behaviours, Shifting Mindsets: Psychological Relief Without Behavioural Change; (c) Disrupted Supply: Dealer Dynamics and Emerging Drug Market Risks and (d) Return of Risk: Heightened Harms following Recriminalization.

### Enduring habits: drivers of drug use patterns amid policy change

The majority of participants reported no changes in the quantity, type, or frequency of drug use following decriminalization. For many, drug use patterns were deeply habitual and shaped by dependence, personal need, and established routines, factors that decriminalization did not meaningfully alter. As one participant explained, “*I’m an addict, right? I need it [drugs]. So [decriminalization] doesn’t affect [my use patterns] now. Just because you say that’s what I can carry, that doesn’t mean that’s what I need”* (Participant 66, Rural, Cis Man, First Nations, Housed). Others echoed this sentiment, noting that their frequency of use remained shaped by daily routines and physiological needs, as described by one participant, *“Whether the government would like to think they do or not, [they] have no effect on how much I need to use in a day or how much I use in a day”* (Participant 37, Urban, Cis Woman, Black, Precarious Housing). For similar reasons, no participants reported changing the types of drugs they used, irrespective of whether those drugs were included under the decriminalization policy, suggesting, *“[Decriminalization didn’t impact my drug use] because I like the drugs that I use. And the change in the law doesn’t really affect my personal case on that”* (Participant 68, Urban, Female, White, Experiencing Homelessness). Among long-term or polysubstance users, the 2.5 g cumulative threshold was widely perceived as unrealistic and insufficient to meet their needs. As one participant put it, “*Well that’s 2.5 g of every drug that you need, so it’s kind of a small amount, yes, because I use a, well I use a lot. […] For a heavy user it’s not very much*” (Participant 57, Rural, Cis Woman, First Nations, Experiencing Homelessness).

Most participants also reported no change in the locations in which they used drugs following decriminalization. Instead, housing status largely influenced participants’ location of drug use. In particular, many housed participants continued to use in private, familiar spaces such as their own residences, stating, *“I never would [change my location of drug use]. My pattern is to come home. Even if I'm out where I'm purchasing [drugs] or something and people offer me a hoot, I won't do anything until I'm at home, feet on the ground”* (Participant 15, Rural, Cis Woman, White, Housed). For participants experiencing homelessness, they described continued use in public locations or at supervised consumption sites/overdose prevention sites (SCS/OPSs), as they had limited options. One participant described this, saying, “*Usually [I use drugs] in my home or in my tent, I live in a tent, or at an OPS*” (Participant 21, Urban, Cis Man, White, Precarious Housing).

Similarly, purchasing and carrying patterns remained largely unchanged. Carrying patterns were also shaped by pre-existing routines, as well as personal preferences or needs, with participants often carrying enough on them at a time to avoid experiencing withdrawal symptoms. Financial circumstances largely drove purchasing patterns, as the amount participants purchased depended on how much they could afford at a given time. When possible, participants preferred to purchase in bulk as that approach was often more cost-effective and convenient. Buying in bulk allowed participants to ‘split and share’ their drugs, where they typically would pick up more at a time on behalf of their friends or peers.

Participants explained that standard purchasing quantities often exceeded the 2.5 g limit, and that breaking these norms could lead to higher costs, but purchasing over the limit could lead to criminalization saying,

“*When there’s a customer buying something, they’re going to get a half gram, a gram, a half ball, or a ball, you know? So if they get more than a half of a ball, they’re going to pay a lot more money for less as opposed to paying less money for more, and put themselves at risk of being charged or have it taken from them*” (Participant 67, Urban, Cis Man, White, Experiencing Homelessness).

Participants frequently noted that the 2.5 g threshold did not align with typical purchasing habits, which were often shaped by how drugs were commonly packaged and sold, suggesting, “*Most people that are buying larger amounts than a gram, in this case usually like 1.75 or 3.5, which is a half and one ball. It just seems really strange that it's stuck at 2.5 [g]”* (Participant 45, Urban, Cis Man, White, Precarious Housing). Several participants recommended raising the threshold to better reflect drug market supply realities. For instance, one participant recommended: *"I think it [the threshold] should be a little more, but that just goes with how the prices work for us and what we buy and stuff […] It’s an odd number. So you don’t go pick up a 2.5, that’s all*" (Participant 75, Urban, Cis Man, White, Experiencing Homelessness).

A few participants reported deliberately altering their behaviours to comply with the legal limit. For these individuals, the decriminalization policy prompted a more cautious and strategic approach to purchasing and carrying drugs: *“[Decriminalization] did [impact how much I carry], yes. I try to keep it down lower than I usually do, or I’ll carry it in 2.5 g packages”* (Participant 50, Urban, Cis Man, White, Precarious Housing). However, purchasing in smaller quantities to abide by the threshold was described as both inconvenient and more expensive.

### Stable behaviours, shifting mindsets: psychological relief without behavioural change

While participants did not report significant changes in their drug use, locations, carrying, and purchasing patterns, many described a psychological shift, indicating that decriminalization had reduced their fear of arrest for carrying substances. The psychological relief also helped alleviate anxiety and self-stigma surrounding their drug use, as one participant described:

*“[Decriminalization] didn’t change how much I carried, but it changed how I felt about it. I wasn’t so sketched out and worried, and I didn’t feel like I was so bad. It kind of felt like I was part of society for a bit. [...] It’s changed how I felt as a person.”* (Participant 27, Rural, Cis Woman, White, Precarious Housing)

This relief was particularly relevant for individuals experiencing homelessness or residing in precarious housing, as public settings were often their primary or only locations for carrying and using drugs, whereby under decriminalization, they felt less at risk of criminalization. For instance, one participant said, “*I did feel a little more comfortable knowing that I wasn’t going to get in trouble for [public drug possession]"* (Participant 58, Rural, Cis Woman, White, Precarious Housing).

In contrast, a smaller number of participants described an opposite experience, stating that decriminalization did not lead to increased psychological comfort regarding their drug use. A small number of participants explained that their addiction had taken over their lives to such an extent that they no longer cared how others viewed them, and as a result, decriminalization had no meaningful impact on their psychological comfort: “*No [decriminalization didn't impact my drug use], I still use the same amount, I don't care*" (Participant 38, Urban, Cis Woman, White/Metis, Housed).

Instead, persistent stigma, particularly as it related to using drugs in public settings, continued to shape their feelings and behaviours. These participants emphasized a strong desire to avoid being seen using drugs in public, suggesting social judgement remained a powerful deterrent. As one participant explained: “*I just kind of don’t want to be seen, number one. And I don’t want to have that kind of negative stigma or connotation following me with that, so I would never do [drugs] outside*” (Participant 51, Urban, Cis Man, White, Housed).

However, among participants who described feeling an increased sense of psychological comfort, most emphasized that this did not translate into more frequent drug use in public settings. Instead, they continued to use drugs in the same spaces they had prior to the policy’s implementation. This finding stands in contrast to the dominant public discourse suggesting that decriminalization has led to a rise in visible public use. While some participants acknowledged these public concerns, they attributed them not to an actual increase in public use, but rather to heightened public awareness and scrutiny following the policy’s implementation. As one participant observed, “*I think when decriminalization first came out, it sort of put public usage in the limelight. And so people began noticing, not that there was an increase in public usage, it’s just people are watching out for it more and reporting it more”* (Participant 22, Urban, Cis Woman, First Nations, Precarious Housing). Others noted that public drug use was often viewed as socially inappropriate or discouraged within peer networks. As one participant described, *“I think that even from when I was on the streets, there is sort of an etiquette around [public drug use] for the most part. People don’t just light up, you know what I mean?”* (Participant 48, Urban, Cis Man, White, Housed).

Several participants further explained that they continued to remain cautious about where they used drugs, and when possible, preferred to use in environments such as an SCS/OPS. As one participant explained:

*“I just felt more comfortable that this [decriminalization] law was keeping us safe. My friends were kind of bugging me to kind of use in public, but I'm like guys, we shouldn't really be doing this in public. It’s not safe. We ought to be at the OPS or a safe injection site or whatever.”* (Participant 47, Rural Cis Woman, White, Precarious Housing)

### Dealer interactions and drug supply: dealer dynamics and emerging drug market risks

While decriminalization resulted in notable psychological benefits for many participants, several reported important changes affecting dealers and the broader drug supply. Participants shared how decriminalization led to disruptions in trusted dealer relationships and a rise in low-level, inexperienced dealers, which they associated with an increase in drug potency and contamination.

Most participants reported that they relied on established dealer relationships as a form of harm reduction, emphasizing the importance of trust, consistency, and the perceived safety of the drugs they received: *"Yes. I mean, I trust my dealer and there’s also other dealers here that I will not, you know. I don’t trust them at all, but the ones that I go to, I do trust"* (Participant 66, Rural, Cis Man, First Nations, Housed). These relationships continued to remain as critical safeguards against the unpredictability of an increasingly toxic drug supply during decriminalization.

However, a few participants observed that their dealers became more cautious and increasingly difficult to access following decriminalization, driven by concerns that enforcement efforts had instead refocused on targeting distributors and suppliers:

*“A few [dealers] have expressed feeling a little bit more cautious about behaving as a distributor because it seems like decrim. We’re lenient towards individuals who use drugs so that we can focus our [police] resources towards stopping the people who sell them.”* (Participant 23, Urban, Transgender Woman, White, Housed)

Some participants noted that dealers were adapting their distribution practices, and believed they were doing so to stay within the 2.5 g legal limit under decriminalization. Specifically, they described shifts in the way drugs were prepared and sold, including a heightened concentration of active ingredients, or increased use of “cutting” the drugs into smaller quantities to make them more potent while maintaining profit. These adaptations were understood by participants as deliberate strategies dealers used to minimize legal risk, allowing them to continue operating within the legal possession limit while offering more potent products. As one participant described: “*[Dealers] would pick up a lower amount and then cut it to be more [potent] than what it was*” (Participant 46, Rural, Cis Woman, Metis, Precarious Housing). Another echoed this concern, linking the increased potency of drugs directly to the threshold, stating, “*It’s the dealers, right? Because they can’t carry as much and so it’s [drugs] stronger*” (Participant 67, Urban, Male, White, Experiencing Homelessness). Some participants elaborated on the dangers of this shift, noting that fentanyl was often intentionally being cut with hazardous substances, which increased the risk of overdose:

“*Yes [the drug supply changed], just specifically with the fentanyl, just different dangerous cuts being added [by dealers]. Xylazine showed up and then heavier benzos showed up. So people were having a lot of benzo withdrawal seizures in this community.*” (Participant 46, Rural, Cis Woman, Metis, Precarious Housing)

Additionally, a few participants were concerned that these changes in drug potency and unpredictable compositions were also attributed in part to a rise in low-level or inexperienced dealers seeking to capitalize on increased demand following decriminalization, suggesting, “*I wouldn’t call them dealers, but yeah, just more people trying to make a buck*” (Participant 75, Urban, Cis Man, White, Experiencing Homelessness). This influx of inexperienced sellers was perceived to have contributed to a decline in drug quality and increased safety risks, as many lacked the skills to properly “cut” or manage drugs, heightening the likelihood of contamination and overdose:

*“My only concern is what [dealers are] putting in [the drugs] that’s not fentanyl. The tranq and those sorts of things. […] But it’s hard to when they’re buying on the streets […] I think [decriminalization has] brought more [dealers] out just because people are – I’m not sure why, but it just seems like there’s a lot more dealers than there used to be.” (*Participant 50, Urban, Cis Man, White, Precarious Housing*).*

### Recriminalization and the return of risk: heightened harms following recriminalization

Following the recriminalization amendment, most participants reported that their drug use practices, including the type of drugs used, amount carried or consumed, the frequency of use, and purchasing patterns, largely remained stable. This continuity mirrored their earlier experiences following the introduction of decriminalization, which similarly had little impact on their day-to-day use, stating, "*[My drug use patterns] didn’t [change since recriminalization]. I carried the same amount before and I’m going to carry the same amount after*" (Participant 62, Urban, Cis Woman, White, Housed). Others emphasized that the amendment had little bearing on their behaviour because they rarely encountered law enforcement and drug use occurred primarily in private spaces. As a result, they perceived the amendment as targeting public drug use and felt largely protected from its implications, with one participant stating, “*[Recriminalization] hasn’t really affected how much I carry because I haven’t had any interactions with the police. I try not to use [drugs] out in public”* (Participant 40, Urban, Cis Man, White, Housed). Participants described this sense of protection often stemmed from using drugs in spaces perceived as safer or less visible, such as private residences, or SCS/OPS: *“I'm not like walking down the street just doing it, I'm like, I'm in someone's house, you know”* (Participant 17, Urban, Cis Man, White, Experiencing Homelessness). They insinuated that the amendment would primarily affect individuals who are more visible to police. This sense of security was especially common among participants who felt more insulated from police profiling, an enforcement practice they suggested often disproportionately targets individuals with more visible indicators of disadvantage:

*“Well, I think [recriminalization didn’t affect me] because I don't possess or appear to possess the attributes of what most people consider problematic substance users in communities. So, homeless population, sometimes people make assumptions by how someone presents as whether they have clean attire. So I have the ability to kind of hide in the crowd as a normal person and I don't think I would be a target by the police.”* (Participant 15, Rural, Cis Woman, White, Housed)

Participants consistently emphasized that the recriminalization amendment disproportionately impacted those who were experiencing homelessness or living in precarious housing, as they lacked private spaces where drug possession remained decriminalized. For instance, one participant described,

“*Well there’s a fairly large homeless population here in [rural location]. And a lot of the homeless people are addicts, and so they’re kind of forced to smoke in public. They don’t have a place to live so they don’t have a private place to use, so they’re kind of forced to use [drugs] in public*” (Participant 58, Rural, Cis Woman, White, Precarious Housing).

Others described feeling increasingly uncomfortable and stigmatized by the public for their use following recriminalization: *“When recrim[inalization] took place, [public drug use] felt a lot less comfortable, but we were still doing it anyway. […] There were no other options”* (Participant 8, Rural, Non-Binary, White, Housed). Most participants further discussed how recriminalization contributed to increased societal and self-stigma, pushing them to engage in risky drug use:

*“People have to hide [following recriminalization] [...] when people drive by, they yell out graphic things to drug users and yeah, people are just hiding now because they don’t want to get their stuff taken from them for the cops. Then that’s why people are dying because they do it by themselves now because they have to hide.*" (Participant 69, Urban, Cis Man, First Nations, Precarious Housing)

Many participants expressed concern that recriminalization was driving people to use drugs in isolation to avoid police encounters, thereby increasing the risk of fatal overdoses, in part due to the absence of bystanders who could intervene. For instance, the following participant stated:

*“I was outside in public with my friends using and then one day there was an incident where we all got told by the cops that we would be charged if we smoked in public. And we stopped hanging out together at the spot. So it promoted less safe use because if you're not hanging out together in a group, you can't Narcan somebody. So it's dangerous to separate people and put them indoors.”* (Participant 42, Urban, Cis Man, White, Housed)

Additionally, some participants described feeling compelled to conceal their drug use to avoid criminalization, which led to riskier behaviours such as reusing drug use equipment or rushed consumption. With the increasing discomfort of using drugs in public, participants sought alternatives, but noted that SCS/OPS were often limited, particularly in rural and remote communities, stating, “*There’s only one OPS in [rural location]*” (Participant 27, Rural, Cis Woman, White, Precarious Housing).

As a result, participants urged the government to create safe, accessible alternatives, as they felt there were limited services available to them:

*“If you don't want people using in public, maybe [the government should] accommodate more places that they can use. Because of our homeless number, it kind of leaves them in a bind, right? Where are they supposed to use then.”* (Participant 9, Rural, Cis Woman, Metis, Housed)

This lack of accessible harm reduction infrastructure was further compounded by confusion surrounding the recriminalization amendment itself. Several participants expressed frustration with the ensuring legal ambiguity, particularly around how they were expected to safely transport their drugs to designated safe-use spaces such SCS/OPS:

*"Yes, [recriminalization] didn't affect how much I carry but it does affect how I carry it. Because I'm concerned, like I said earlier, if it's decriminalized to have possession but only in an OPS or at home, then how do I get to the OPS? Are they going to jack me on the way to the OPS or right outside the OPS? When I'm standing in line waiting to get into this OPS?"* (Participant 38, Urban, Cis Woman, White/Metis, Housed)

Similar to experiences during decriminalization, where participants noted disruptions to their access to trusted dealers, several participants described how this effect was significantly worse under recriminalization. Another heightened risk following the recriminalization amendment was the disruption of established drug purchasing networks. Increased enforcement activity, such as heightened police presence, arrests, or displacement of known dealers, undermined participants’ ability to access drugs through trusted, consistent sources. When these networks were destabilized, participants were often forced to seek out unfamiliar or low-level dealers, where product quality was uncertain and the risk of adulteration or overdose was higher:

*“Before decriminalization I had always really been buying from the same people, and recriminalization came in, I couldn’t buy from the same people anymore because they were busted, or they were selling less [...] and I had to go look around other places. And it’s a scary thing [...] I would say that it did raise my chance of overdose quite a bit because I was buying from people that I didn’t necessarily know very well yet.”* (Participant 1, Urban, Cis Woman, Metis, Precarious Housing)

In some cases, participants reported dealers becoming more cautious during drug purchases, which caused them to change the locations of deals or scale back their sales:

*“Now that it’s recriminalized again, I am a little more cautious where I go because of police, and I try to avoid [them]. So sometimes I go to the more shady places where you can go into a back window and kind of get your drugs. And those places are a lot more dangerous to buy drugs at because those people do not know you on a personal note, like most of the other dealers in this small community where they’re not going to give you bad, tainted stuff.”* (Participant 18, Rural, Cis Man, White, Housed)

These shifts in dealer behaviour not only disrupted access to trusted sources, but pushed participants toward more dangerous environments and unfamiliar suppliers, further compounding the risks associated with an increasingly unstable drug market.

Most participants therefore viewed the recriminalization amendment as a significant barrier to the overall success of the policy. They viewed the short-term nature of decriminalization combined with the abrupt policy change as “breaking the trust” that people who use drugs had with the government, suggesting,

*“If the initial policy is still being carried out, like we're still in the two year, but now they're re-criminalizing something, that's breaking the trust. Like people don't know what's going on either. I just heard through the grapevine it was re-criminalized, and that's just scary. So it just makes you more paranoid.”* (Participant 14, Urban, Cis Woman, White, Housed)

## Discussion

While BC’s decriminalization policy did not significantly change drug use behaviours, as most participants reported continuing to use, purchase, and carry drugs in the same ways, it nonetheless had important impacts, as many described a sense of psychological relief following the policy’s implementation, including reduced, anxiety, stigma, and fear of arrest associated with drug possession. This psychological shift was meaningful for many people who use drugs, as it fostered a greater feeling of safety in their daily lives. However, despite an alleviation of concern around using, purchasing, and carrying drugs, some participants noted unintended consequences of the policy on the drug supply. Taken together, these findings suggest that while behavioural change was limited, the policy may have influenced the broader social and structural environment in which drug use occurs.

Our findings suggest that decriminalization, in isolation, did not naturally transform drug use behaviour shortly after the policy implementation. Instead, patterns related to frequency, amount, substance type, and carrying and purchasing remained largely unchanged following decriminalization. For many participants, daily consumption and drug use related patterns were governed less by legal considerations and more by psychological and physical needs like withdrawal management, as well as by logistical concerns including access to trusted suppliers or financial constraints. Rather than reducing rates of use, existing research suggests that decriminalization primarily reduces the harms associated with criminalization, such as fear, stigma, and barriers to health and social services [27]. The fact that drug use behaviour remained unchanged highlights the enduring influence of structural determinants of health, such as poverty, housing instability, trauma, systemic racism, and service access, that shape drug use patterns and constrain the ability or desire of individuals to alter behaviours in response to legal reform [28]. These findings underscore a core disconnect between the policy, and the complex realities of substance use, particularly among long-term or polysubstance users, whose patterns are to carry larger amounts and various types of substances for the change in policy to affect their needs without experiencing withdrawal symptoms.

While the changes in drug use behaviours were limited among study participants, the implementation of decriminalization offered a sense of psychological relief from the fears of criminalization. Even in the absence of behavioural change, participants described feeling less fearful, fostering a sense of personal safety in their day-to-day lives. The policy’s symbolic departure from criminalization signaled to many that they would not be punished simply for their drug use. These findings are consistent with international research examining drug policy shifts across 20 countries, which has shown that moving from punitive, prohibitionist, criminalized frameworks to more liberal, health-focused approaches, such as decriminalization, can reduce fear of arrest among people who use drugs, self-stigma, and subsequently foster engagement with health and social services [29]. These narratives reinforce findings from Portugal’s long-standing decriminalization model, where removing criminal penalties produced gradual but significant reductions in both societal and self-stigma [29–31]. However, Portugal’s longstanding decriminalization model was accompanied by major structural reforms [31], while participants in our study noted that BC still lacked sufficient harm reduction and treatment services. When individuals are not constantly anticipating surveillance or criminal charges, they are more likely to seek support, access harm reduction supplies, and engage in care [27, 32–34]. BC’s decriminalization framework, though limited in scope, thus allowed participants to feel “safer” when carrying drugs, and less hypervigilant in public spaces, providing a psychological reprieve from the fear of criminalization.

However, this sense of relief was tempered by the practical limitations of the policy, particularly the disconnect between the 2.5 g cumulative threshold for personal possession and the realities of drug use. The policy was designed with engagement from people with lived and living experiences, however, the threshold has been widely perceived as arbitrary and misaligned with lived and living experiences, and real world practices. This raises concerns about whether meaningful engagement was involved, as the threshold ultimately failed to reflect established consumption and purchasing patterns. Research has shown that low thresholds can undermine the goals of decriminalization by disregarding structural inequities and everyday practices of people who use drugs [34]. As a result, harm reduction organizations and advocacy groups have long called for the threshold to be either removed entirely or raised from anywhere to 3.5 to 28 g, stating that 2.5 g does not align with the needs of people who use drugs [36]. These calls have been backed by research, highlighting that 2.5 g is not sufficient for many people who use drugs [14], and a higher threshold would better align with the policy’s goals of reducing criminal penalties for personal possession, taking into account the needs of marginalized groups, as well as long-term, high frequency users, who typically purchase and carry above this amount [35]. For high-frequency users, long-term users, and those living in rural and remote areas who often travel long distances and purchase in bulk, the 2.5 g cap was especially unworkable [35, 37]. While intended as a safeguard, the threshold paradoxically introduced new risks by discouraging bulk purchasing and disrupting trusted dealer relationships, both of which are established harm reduction strategies [14]. As a result, participants described increased exposure to riskier, more frequent transactions and a shift toward less reliable sources, echoing findings that consistent dealer relationships can act as informal safeguards against overdose and contamination [38]. For instance, research has shown that trusted dealers may act as informal regulators, testing drugs or refusing to distribute substances known to be adulterated with fentanyl or other dangerous additives such as tranquilizers, benzodiazepines, and/or nitazenes [38, 39].

Further, this disruption in dealer relationships directly impacted the drug supply itself. Participants described how, in an attempt to comply with the 2.5 g threshold while preserving profits, some dealers began increasing drug potency, by “cutting” substances with more powerful or dangerous additives, thereby introducing greater variability and toxicity into the supply. These concerns were not merely anecdotal, as participants described noticeable increases in the strength of fentanyl, which, along with the growing presence of tranquilizers and sedatives, further complicated overdose prevention and response, as these drugs do not respond to naloxone [40]. These dynamics align with the ‘iron law of prohibition,’ a well-documented phenomenon in drug policy, which suggests that as drug enforcement intensifies, suppliers respond by increasing drug potency to preserve profit while reducing the actual amount of drugs being sold [41, 42]. This has been observed in multiple jurisdictions, where market pressures, including intensified policing and supply disruptions, contribute to the proliferation of highly potent synthetic opioids, such as fentanyl and its analogues. In these settings, increased enforcement efforts have led to a greater unpredictability in drug potency and a toxic, unstable supply that dramatically increases overdose risks [43].

While some participants attributed changes in the drug composition and potency to the implementation of BC’s decriminalization policy, it is important to interpret these perceptions within a broader national context. Shifts in drug supply, including increased potency, unpredictable mixing of substances (e.g., benzodiazepines, nitazines), and changes in product appearance and naming, have been documented across multiple provinces in Canada outside of BC. For instance, data from Ontario, Alberta, and Quebec indicate growing contamination of the unregulated drug supply with synthetic opioids and non-opioid adulterants, independent of decriminalization efforts [44, 45]. The emergence of novel substances like benzodiazepines and xylazine has also been reported nationally [45], suggesting that the observed changes may reflect broader shifts in the unregulated market, driven by factors such as supply chain dynamics, border enforcement, and profit maximization by suppliers, rather than being specific consequences of BC’s policy. Therefore, while participants’ perceptions offer valuable insight into how policy and market conditions are experienced on the ground, these changes in drug composition and supply are part of a complex, evolving national trend and not necessarily a direct outcome of decriminalization. This distinction is important, as it underscores the need for national strategies to monitor and respond to the toxic drug supply, alongside localized policy responses like decriminalization.

Furthermore, the recriminalization amendment was also reported to have significantly disrupted participants’ drug use environments, particularly for those without access to private, indoor spaces. However, the amendment reversed many of these perceived gains in safety and dignity, especially for those who relied on public spaces. Participants described an urgent need to ‘use and hide’, which often pushed them into more isolated and hazardous environments, such as alleyways, stairwells, tents, or behind dumpsters. These settings are among the most well-documented risk factors for fatal overdose, as they significantly reduce the likelihood of timely intervention, including naloxone administration, or emergency response, and put people who use drugs at increased risk of physical and sexual violence [11, 46–48]. Similar patterns have been documented in previous research, where increased police presence and enforcement activities led to ‘rushed’ injections, discouraged safer injection practices, and displaced people who use drugs from harm reduction services [49].

Critically, these risks were not experienced equally across participants. A clear divide emerged between housed participants and participants experiencing homelessness. Re-criminalization amplified environmental risks for participants experiencing homelessness, who experienced intensified policing in public spaces, despite having no access to safer private locations. These dynamics illustrate the structural inequality embedded in the enforcement of drug policy and highlight the need for environmental-level interventions, not just behavioral ones. Those with stable housing often reported minimal fear of police interaction, attributing this to their ability to use indoors and a sense of being shielded by their physical appearance, housing status, and ability to use indoors, what some researchers have referred to as “privileged invisibility” [50]. These individuals felt insulated from scrutiny and able to continue their routines without significant fear of enforcement. However, this underscores a disconnect between one of the goals of the policy—reducing the risk of overdose by discouraging solitary use—and the lived realities of housed people who use drugs. Many housed participants continued to use drugs in private residences, where they may still face heightened risks for fatal overdose, rather than accessing supervised services, such as SCS or OPS. In contrast, participants experiencing homelessness expressed heightened vulnerability and fear, noting that their visibility, location, and perceived status as street-involved made them more likely to be profiled, surveilled, or targeted by law enforcement. This was further aggravated by the amendment, leaving very few safe, ‘decriminalized’ spaces available for people who use drugs and are experiencing homelessness to use their drugs without fear of arrest [51].

This inequitable impact reflects longstanding critiques of drug policy enforcement as unequally applied, reinforcing racialized, classed, and spatial hierarchies [52]. The recriminalization amendment intensified this divide, reinforcing the structural inequities that shape whose drug use is punished and whose is ignored. These findings raise serious concerns about the unintended consequences of the policy reversal. For populations already at high risk of overdose and exclusion from services, especially individuals experiencing homelessness, the erosion of safe, non-criminalized spaces may further increase health risks and undermine the broader goals of drug decriminalization. As such, any future drug policy reform must be accompanied by clear protections for the most marginalized, and a recognition that legal changes without structural support may ultimately reproduce the very harms they aim to prevent.

## Limitations

Participants came from a wide range of geographic regions and backgrounds, and represented diverse substance use patterns and lived experiences. However, these findings are not intended to be representative of all people who use drugs in BC. Recruitment strategies, which were largely facilitated through harm reduction services and advocacy organizations, may have resulted in a sample who are more integrated and connected to service networks, which could differ meaningfully from those who are more isolated or disconnected from formal supports. Additionally, social desirability bias and recall bias may have also influenced participant responses. Given the stigmatized nature of the interview topic, social desirability bias may have led participants to underreport certain aspects of their drug use or involvement with the criminal justice system. Since participants were interviewed during recriminalization, recall bias may have influenced participants’ view of decriminalization. Specifically, they may hold a more positive view of decriminalization in retrospect.

Moreover, while participants often spoke from personal experience, some responses reflected more generalized observations of their communities or peers, rather than direct individual impacts of the policy. Finally, while self-reported demographic data were collected, the present study did not fully disaggregate findings across intersecting identity categories beyond basic reporting of rural and urban location, housing status, gender, and ethnicity. These intersecting factors are likely to influence how drug policy is experienced, and future research should explore these dimensions in greater depth.

## Conclusion

Decriminalization had little effect on drug use patterns, as the frequency, quantity, and type of drugs participants purchased and used were largely shaped by structural factors, such as economics, and longstanding routines and habits. However, decriminalization fostered a notable psychological shift where participants reported feeling less stigmatized for their drug use. The reintroduction of criminal penalties for using in public ultimately reversed many of these early gains and reimposed conditions of risk, forcing people who use drugs into hidden, unsafe spaces to use drugs. Although the amendment was established as a means to protect public safety, participants perceived it as counterproductive, heightening the harms that decriminalization sought to mitigate. Ultimately, the recriminalization amendment underscores the complexity and unintended consequences of abrupt policy reversals, which can deepen existing harms and further marginalize people who use drugs.

## Data Availability

The datasets generated and/or analyzed during the current study are not publicly available to protect study participant privacy.

## Abbreviations

BC: British Columbia
CAMH: Centre for addiction and mental health
CHSA: Community health service areas
CIHR: Canadian Institute of Health Research
CRISM: Canadian research initiative in substance matters
OPS: Overdose prevention site
REB: Research ethics board
REDCap: Research electronic data capture
SCS: Supervised consumption site
